# Myc as a Regulator of Ribosome Biogenesis and Cell Competition: A Link to Cancer

**DOI:** 10.3390/ijms21114037

**Published:** 2020-06-05

**Authors:** Francesca Destefanis, Valeria Manara, Paola Bellosta

**Affiliations:** 1 Department of Cellular, Computational and Integrative Biology (CiBio), University of Trento, 38123 Trento, Italy; francesca.destefanis@unitn.it (F.D.); valeria.manara@studenti.unitn.it (V.M.); 2 Department of Medicine, NYU Langone Medical Center, New York, NY 10016, USA

**Keywords:** Myc, *Minute*, cell competition, ribosome biogenesis, ribosomal proteins, growth, cancer, *Drosophila*

## Abstract

The biogenesis of ribosomes is a finely regulated multistep process linked to cell proliferation and growth—processes which require a high rate of protein synthesis. One of the master regulators of ribosome biogenesis is Myc, a well-known proto-oncogene that has an important role in ribosomal function and in the regulation of protein synthesis. The relationship between Myc and the ribosomes was first highlighted in *Drosophila*, where Myc’s role in controlling Pol-I, II and III was evidenced by both microarrays data, and by the ability of Myc to control growth (mass), and cellular and animal size. Moreover, Myc can induce cell competition, a physiological mechanism through which cells with greater fitness grow better and thereby prevail over less competitive cells, which are actively eliminated by apoptosis. Myc-induced cell competition was shown to regulate both vertebrate development and tumor promotion; however, how these functions are linked to Myc’s control of ribosome biogenesis, protein synthesis and growth is not clear yet. In this review, we will discuss the major pathways that link Myc to ribosomal biogenesis, also in light of its function in cell competition, and how these mechanisms may reflect its role in favoring tumor promotion.

## 1. Ribosome Biogenesis 

Ribosomes are the molecular machines responsible for decoding mRNAs into proteins. In eukaryotes, the ribosome is composed of the 40S and 60S subunits that associate to form the translationally active 80S ribosome. Eukaryotic 80S ribosomes are made of 4 ribosomal RNAs (rRNAs) and 80 core ribosomal proteins (RPs). The main difference between eukaryotes, protists and yeasts rely on the modest increase in the overall protein mass of ribosomes. In particular, the *Drosophila* 80S ribosome is made of 1094 amino acids, whereas the human 80S is composed of 796 amino acids, accounting for 6% difference in their protein mass [1]. The coding sequences of the RP genes in humans and *Drosophila* are highly conserved and share 69% homology. Human ribosomal proteins genes are 4–5 times larger than those in *Drosophila,* since the introns in the human sequences are bigger in size and in number. Moreover, among the 249 introns found in the coding regions of the ribosomal protein genes, 77 share the same insertion sites in humans and flies [2].

**Figure 1:**
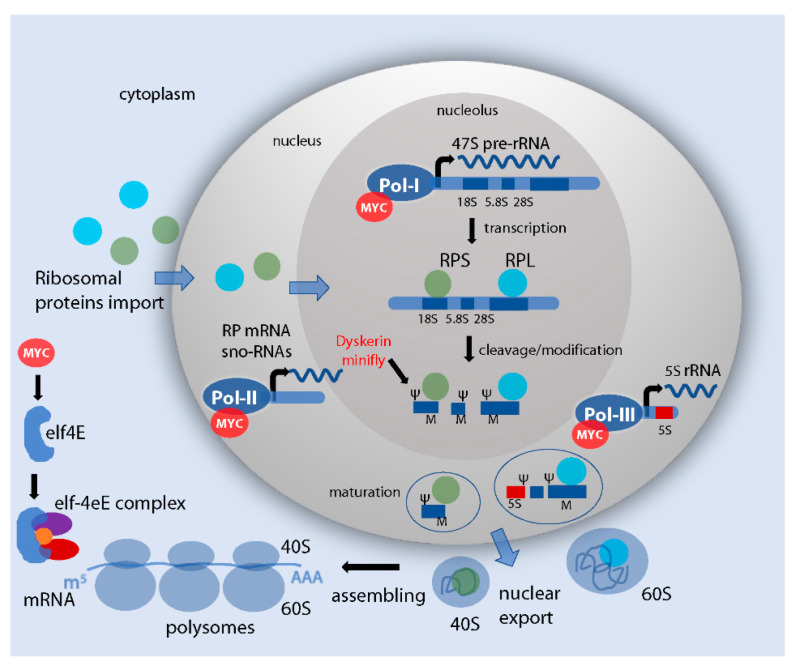
Control of ribosome formation and RNA translation. Schematic representation of the essential steps in ribosome biogenesis in humans and of the relative regulation by Myc (in red). In the nucleolus, interacting with the complex Pol-I, Myc increases rDNA transcription and the formation of pre-rRNAs. The pre-rRNA is then processed and cleaved to form the 18S, 5.8S and 28S. These RNAs are assembled with ribosomal proteins (RPs) with the assistance of snoRNAs that mediate important RNA modifications—in particular, small nucleolar RNAs belonging to those of box C/D mediate O-methylation (M) and to those of the box H/ACA pseudourydilation (Ψ). In these steps, Myc activity controls Pol-II for the transcription of snoRNAs and specific RPs. Of note: in *Drosophila,* Myc was shown to directly control the expression of the snoRNP dyskerin (in flies called *minifly*, reviewed in 2.3, due to its phenotype that is reminiscent of that of *diminutive* mutants, reviewed in 2.1). The pre-assembled ribosomal units form the premature 40S and 60S subunits in the nucleolus. In the nucleus, the activity of Pol-III to encode for the 5S rRNA, necessary for the proper maturation and the assembly of the 60S subunit, has been associated with Myc activity. The two mature subunits are then exported into the cytoplasm and assembled into mature ribosomes or polysomes, ready to perform translation of mRNAs and protein synthesis. In the cytoplasm, the translation is initiated with the formation of the initiation factor 4F (eIF4F) complex, that includes the cap-binding protein eIF4E, the scaffolding protein eIF4G (in purple) and the helicase eIF4A (in red) [14]. Myc in humans promotes the translation of eIF4F, and cooperates with elF4E to drive tumorigenesis in vivo [15].

The process of making ribosomes is tightly coupled with cell proliferation and growth. It is a complex biological mechanism that involves multiple coordinated steps and requires the synthesis, processing, and assembly of different proteins and RNA components. This process is initiated in the nucleoli, then continues in the nucleoplasm, followed by the export of precursor particles to the cytoplasm, where the complete assembly takes place (Figure 1). Nucleoli are intranuclear compartments which assemble around the tandem repeats of ribosomal genes that organize ribosomal DNA (rDNA), which encodes the 5S, 5.8S, 18S and 28S rRNAs in eukaryotes. The nucleolus is divided in different subregions, each of them specialized in specific steps for the formation of the ribosome subunits, in which the fibrillar centers (FCs), the dense fibrillar components (DFCs), and the granular components (GCs) are visible. Transcription of the rDNA repeats occurs largely at the border between the FC and DFC. The processing and modification of the pre-rRNA transcripts occurs in the DFC where small nucleolar ribonucleoproteins (snoRNPs) accumulate, whereas most proteins concentrate in the GC, where ribosome subunit assembly is completed [3,4,5,6,7].

In the nucleolus, the initial transcription of rDNA genes is mediated by RNA polymerase I (RNA Pol-I), which in humans transcribes a single 47S rRNA precursor, that is subsequently cleaved to form mature 28S, 18S and 5.8S rRNAs [8]. The 5S rRNA is encoded by tandem arrays in chromosome regions outside the nucleolus and it is transcribed by RNA Pol-III [9]. rRNAs are then post-transcriptionally modified to introduce a methyl group at the 2-O position of the ribose sugar residues and pseudouridines. These modifications are mediated by the interaction with snoRNPs, belonging to the box C/D (for O-methylation) and box H/ACA-snoRNPs (for pseudouridylation) [10], and other protein-processing factors responsible for the majority of rRNA modifications [11,12,13].

Ribosome biogenesis also requires the transcription of two classes of ribosomal proteins, whose translation is mediated in the cytoplasm by RNA Pol-II. RPs are then imported into the nucleus, where they are assembled into small and large ribosomal subunits. The small 40S ribosomal subunit contains one 18S rRNA and 32 ribosomal proteins (known as RPS), whereas the large 60S subunit is composed of one of each 5S, 5.8S and 28S rRNA and 47 ribosomal proteins (known as RPL). The 40S and 60S subunits are then exported into the cytoplasm, where they assemble with mRNA to form the 80S ribosome [3,4,6].

## 2. Role of Myc in Ribosome Biogenesis

A well-known master regulator of ribosome biogenesis is Myc, a proto-oncogenic transcription factor, which controls the transcription of at least 15% of the human genome and has been described as a global regulator of several cellular processes, including the regulation of chromatin structure, translation, DNA replication and cellular differentiation [16]. In vertebrates, the Myc-network is composed of the three transcriptional activators of the Myc family (c-, N-, L-Myc), the several transcriptional repressors of the Mad-family, and the common dimerization partner Myc-associated factor X (Max) [17]. The vast majority of Myc targets are involved in ribosome biogenesis, including components that boost the activity of Pol-I, II and III simultaneously [18,19]. This is not surprising, considering the need for increased protein synthesis, and thus ribosome biogenesis, necessary to sustain the higher proliferative rate and growth that are often seen with Myc expression.

### 2.1. Drosophila Myc and the Regulation of Size

In flies, the Myc-network consists of a single transcriptional activator (Myc), a single repressor (Mnt), the orthologue of the repressor of the Mad-family, and the common dimerization partner Max [20]. As in vertebrates, Myc acts as a heterodimer with Max, and the complex Myc:Max directly binds E-box sequences (CACGTG), located at specific positions in the promoter regions of the target genes [21,22] to recruit co-regulatory factors, including members of the chromatin remodeling complexes and the Tip48/49 ATP/helicases, in a mechanism that is conserved also in vertebrates [23,24]. The repressor Mnt can bind Max and recognize the E-box sequences, antagonizing the effects of the Myc:Max complex. Several Myc-activated target genes are indeed repressed by Mnt, suggesting that Myc and Mnt have opposite roles in regulating cell growth [25,26,27,28,29,30].

**Figure 2:**
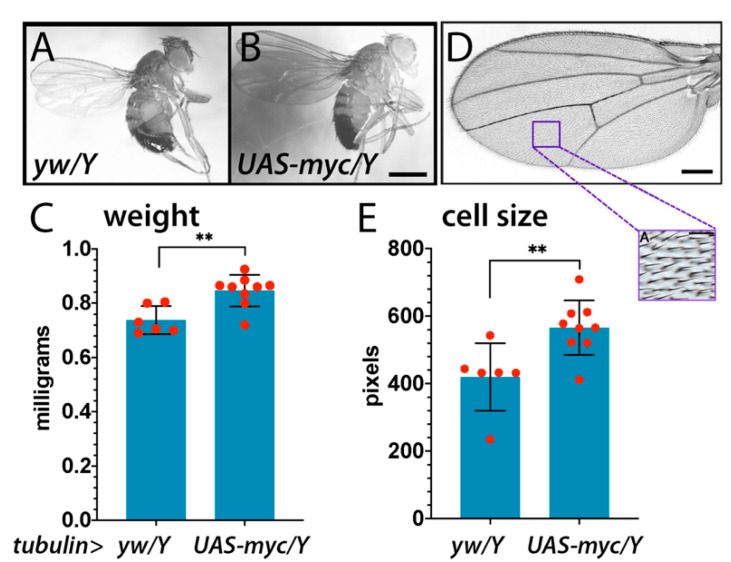
Myc ubiquitous expression increases animal weight and cell size. (**A**–**C**) Expression of Myc using the ubiquitous promoter tubulin (*tubulin* > *Gal4*; *UAS-Myc* [33]) significantly increases body size and weight. (**A**,**B**) Photographs of control *yw/Y* males and animals expressing Myc ubiquitously (*UAS-Myc*) using the *tubulin-Gal4* promoter; scale bar 1 mm. These flies were weighed, and the results are represented in the graph (**C**); as shown, expression of Myc increases body weight. (**D**,**E**) Myc expression increases cell size. The cell size was analyzed in the adult wing by measuring the number of the trichomes present in the wing blades of animals of the different genotypes (control *yw/Y* males and animals expressing Myc ubiquitously using the *tubulin-Gal4* promoter). Each cell of the epidermal surface of the wing carries a protrusion called trichome, that is visible in the adult wing. The number of trichomes is then equivalent to the number of cells from the epithelium. By considering a specific area of the wing (indicated by the square), and by measuring the number of trichomes present in the fixed square, it is possible to analyze the density of the trichomes, which is indirectly proportional to cell size (the higher the density, the smaller the cell size); scale bar 200 μm. (**E**) Graph representing the calculated cell size from experiments in (**D**). Taking into account that the lower the number of trichomes in each square the larger is the cell size, these experiments concluded that expression of Myc increases the size of the animal because it increases the cell size, and not because it induces proliferation. The asterisks represent the *p*-values from *t*-test student ** = *p* < 0.01, the error bars indicate the standard deviations, the number of animals is indicated by the red dots. The same conclusion was observed in females.

Interestingly, a fly mutation called *diminutive* (*dm*), that phenotypically affected the organismal size, was isolated in *Drosophila* in 1935 by Calvin Bridge. Only lately this mutation was molecularly characterized, and it corresponded to a hypomorphic mutation of the *Drosophila myc* gene, whose sequence shares a moderate 26% overall homology with its human counterpart *c-myc* [26,31]. Some hypomorphic *dm* alleles were identified and characterized. The first mutant was called *dm*^*P*0^ and showed defects at both organismal and cellular levels, resulting in decreased cell size, body size and viability [32]. In 2004, the characterization of a null mutation (referred as *dm*^4^) revealed that the complete loss of Myc further reduced larval growth, and these mutants died during early larval development [27]. In 1999 it was first observed that clones generated in the imaginal discs that overexpressed Myc were bigger than controls, as a result of larger cell size compared to that of wild type cells [32]. Later, ubiquitous overexpression of Myc was shown to have a similar effect, resulting in flies that were overall bigger in size due to an increase in cell size (Figure 2) and not in cell number, suggesting a specific function of Myc in controlling the growth rate [33].

**Figure 3:**
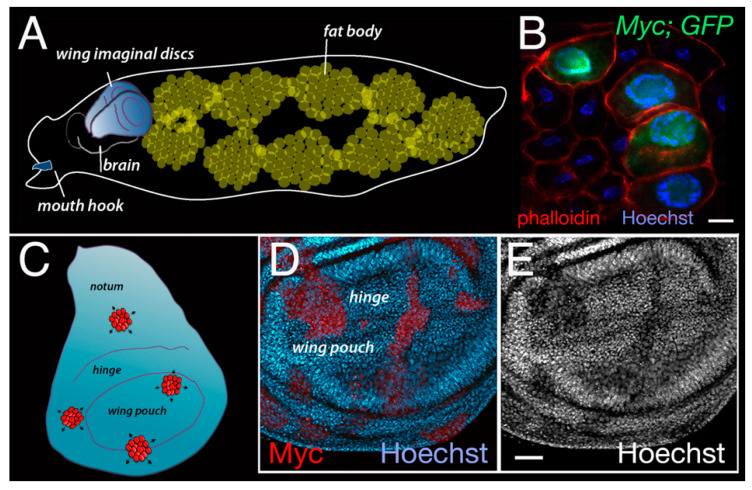
Expression of Myc increases nuclear size. (**A**) A schematic representation of a Drosophila larva indicating the position of the wing imaginal discs and of the fat body. (**B**) Photograph of cells from the fat body showing clones of cells overexpressing Myc generated using the flip-out UAS-Gal4 technique. This method allows the temporal induction of clones using the enzyme flippase under the control of the heat-shock promoter (hsp70). The expression of the flippase is induced with a heat-shock at a specific time of the development to excise the FRT > sequences in the construct actin > CD2 > Gal4; UAS-HA-Myc; UAS-GFP. Gal4 is then produced under the control of the constitutive actin promoter, and binds the UAS sequences upstream of the myc cDNA allowing its expression. UAS-GFP is co-expressed and used as a marker for the clones [35]. Phalloidin (in red) labels the membranes, while nuclei are stained with Hoechst; scale bar 20 μm. (**C**) A schematic representation of a Drosophila wing imaginal disc expressing clones induced using the flip-out technique. (**D**,**E**) Photographs of the pouch of a wing imaginal disc of a third instar larva, showing the presence of flip-out clones expressing Myc (in red). (**D**) Myc protein is visualized by immunostaining using an anti-HA antibody that binds to its tag at the N-terminus [36]. Nuclei are stained with Hoechst (green). (**E**) is the same wing disc as in D, only in black and white to better visualize the nuclei. Myc ability to induce endoreplication [27] results in cells having bigger nuclei, which appear sparse in the photo due to their increase in size compared to nuclei in wild type cells; scale bar 50 μm.

A dramatic effect of *Drosophila* Myc is observed in endoreplicating polyploid cells necessary to build most of the larval mass [27,34]. These cells, such as those of the fat body (Figure 3), gut, salivary glands and ovary, can reach ploidies of up to 1000 n. The effect of Myc on endoreplication is clearly visible in the polyploid cells of the fat body, due to a tremendous increase in their DNA content when Myc is overexpressed (Figure 3B). Myc’s effect on nuclear size is also detectable to a lesser extent in the epithelial cells of the imaginal discs, where its expression results in a massive increase in the nuclear volume (Figure 3D,E) [27]. On the contrary, in Myc mutant animals, endoreplication is strongly impaired and the nuclear volume of most of the cells is reduced. As a consequence, Myc mutant larvae are considerably smaller [27].

### 2.2. Myc Controls the Transcription of rDNA, Ribosomal Proteins and snoRNAs Genes

Beside the induction on endoreplication, the overall effect of Myc’s on cell size can also be generally explained by its ability to promote and sustain ribosome biogenesis (Figure 1), a function that was first evidenced by work in *Drosophila,* supported by microarray and genomic studies, that revealed the importance of Myc in controlling genes involved in the proper formation and activity of the ribosomes [21,22,37]. Both in flies and vertebrates Myc was shown to recognize E-box sequences in the coding regions of rDNA, thus promoting the transcription of 18S, 5.8S and 28S rRNAs [38,39,40].

Myc’s function as a transcriptional regulator was initially recognized for its ability to activate chromatin remodelers and cofactors, allowing for the recruitment of RNA polymerase II on bona-fide Myc target genes [19,41,42]. Microarray and genomic data showed its ability to also control members of chromatin remodeling components, such as TRRAP (Transformation/Transcription Domain Associated Protein), as a part of a histone acetyltransferase complex that increases the acetylation of nucleosomal histones H3 and H4 in the rDNA promoter region, a function that suggests Myc could also control pre-ribosomal RNAs [43].

Outside the nucleolus, Myc in humans is also able to induce the transcription of 5S rRNA by interacting with TFIIB and RNA Pol-III [44,45], and together with the histone acetyltransferase GCN5, can enhance the transcription of tRNAs [43]. Finally, Myc was also found to enhance the recruitment of RNA Pol-I on rDNA by interacting with TATA box-binding protein (TBP) and RNA Pol-I specific TBP-associated factors (TAFs), leading to the activation of Pol-I on rDNA and thus transcription of rRNAs [38,40]. This function is conserved in flies, where Myc was shown to be necessary for the regulation of rRNA synthesis, a rate-limiting passage in cellular growth [37].

Works from Gallant’s group recently characterized and identified snoRNAs that are directly regulated by Myc, and in addition used *Drosophila* cells to identify a family of genes, called U-snoRNA host genes (*Uhg9s*) that are able to control the synthesis of specific snoRNAs [46]. Moreover, this function has also been described in human cells, where Myc was shown to control the SNHG7 loci that encodes for genes that are important in the regulation of snoRNAs in vertebrates, adding another important piece in deciphering the puzzle of how Myc and snoRNAs function in human cancer [47].

As part of this approach to understand the role of Myc in translation and cancer, recently amino acid tRNA synthetases (aaRSs) were identified as necessary for the growth and survival induced by Myc, both in *Drosophila* and in human HMEC cells [48].

All these observations again highlight the ability of Myc to increase and promote cellular biosynthesis, particularly in tumor cells, at the cost of physiological regulation of protein during cellular homeostasis.

### 2.3. Myc Controls rRNA Processing and Assembly of the Ribosomes

rRNA synthesis by RNA Pol-I is a process conserved from yeast to mammals that must be finely regulated in all of its aspects. The initiation complex must be coordinated with other biological processes, including co-transcriptional processing, re-initiation of transcription, and with the mechanisms occurring downstream of RNA Pol-I transcription that ensure the correct maturation and transport of newly formed pre-ribosomes [49]. Myc was shown to control the expression of factors required for rRNA processing including fibrillarin, nucleolin and nucleophosmin [45,50,51,52]. Particularly important is its direct regulation of dyskerin, a bona-fide Myc target that contains an E-box in the promoter region, as this enzyme is responsible for pseudouridylation of targets such as snoRNAs and rRNAs [12]. Moreover, *Drosophila* hypomorphic mutants of dyskerin, also called *Nop60b* or *minifly (mfl)* [53], have been shown to phenocopy the growth defects of *diminutive* mutants, and cells mutant for *mfl* are out-competed via cell competition [54], suggesting that *mfl* is a target of Myc in this process (described in Section 3). These data are important as they support the hypothesis that Myc may control cell competition directly through the regulation of RNA modifications such as pseudouridylation, suggesting a novel function of RNA modification as part of a “ribosomal code” [55], according to which specialized ribosomes are formed that control translation in specific process, i.e. in cell competition.

Along the same lines, our microarray analysis of Myc-target genes identified a family of genes, called *noc1p(s),* that in yeast were shown to be involved in the correct maturation of the large subunit of pre-ribosomes and in their transport within the nucleolus [56,57]. Indeed, work in yeast showed that Noc1p and Noc2p are recruited, along with Rrp5p, to form a transcriptional complex on the nascent pre-rRNA, and after cleavage of the rRNA precursor these factors remain associated with the pre-40S and pre-60S components. Their function is not totally clear; however, mutant strains for Noc1p, Noc2p and Noc3p accumulate pre-60S particles that fail to correctly leave the nucleolus, suggesting they may function in the protection of pre-ribosomal particles from aberrant RNA processing and degradation events [58]. Notably, we found that a few of these *noc1p* genes contain bona fide Myc-binding E-boxes in their promoter regions, suggesting that they may be directly involved in the control by Myc of the pre-RNA maturation process (FD, VM, PB, in submission).

### 2.4. Myc-Dependent Regulation of Ribosome Biogenesis Can Be Influenced by Growth Factors Signaling

The Myc-dependent regulation of ribosomal genes is also controlled by growth factor signaling. Indeed, it is now emerging that Myc can act in response to growth factors availability and nutrient uptake to control rRNA processing. Numerous studies indicate an important cooperation between Myc and TIF-IA, which is the rate-liming transcription factor in the initiation of rRNA transcription, since it is able to connect RNA Pol-I with the upstream binding factor (UBF)/SL-1 (TIF-IB) complex to form the pre-initiation complex on the rDNA promoter [41]. Specifically, it has been shown in *Drosophila* that S6 Kinase (S6K), downstream of TOR signaling, is essential for Myc-dependent rDNA transcription by activating TIF-IA in cooperation with Myc. Loss of S6K leads to a nutrient-dependent reduction in transcription of factors required for rDNA transcription [59]. Moreover, ERK-mediated phosphorylation of TIF-IA, downstream of the Ras signaling pathway, is required for its activation and thus RNA Pol-I transcription. Phosphorylation events downstream of Ras/ERK are also fundamental to post-transcriptionally regulate the stability of Myc, which has a very short half-life (20 min both in humans and flies) [36,60,61]. Thus, the Ras signaling pathway may play a part in rDNA transcription in response to growth factors by cooperating with Myc. TIF-IA can also be phosphorylated by AKT, downstream of the PI3k pathway. In this way, AKT promotes rRNA synthesis and cooperates with Myc to sustain ribosome biogenesis [62]. Furthermore, LKB1 kinase, upstream of AMPK signaling, actively promotes TIF-IA nuclear import in order to maintain rRNA synthesis and prevent cell death under stress conditions [63]. Therefore, it is clear that TIF-IA, Myc, and ribosome biogenesis in general are strongly regulated by kinases that respond to growth factors, amino acids and nutrient status.

Notably, these kinases, which are oncogenic, in cooperation with Myc can trigger a metabolic reprogramming typical of cancer cells, in order to energetically sustain the increased demand for building blocks necessary to increase cell mass and promote DNA replication and cell division [42].

In humans, Myc has been shown to promote the eIF4F-dependent translation, and to cooperate with elF4E to drive tumorigenesis in vivo [15]. Translation initiation is considered a rate limiting step for protein synthesis that proceeds in a cap-dependent manner, with the formation of a complex composed of the initiation factor 4F (eIF4F) together with the scaffolding protein eIF4G and the helicase eIF4A, that are able to bind the 5′-7methylguanosine cap present on mature RNA [14]. This control of Myc has been described also in human cancer cells with the activation of growth signaling pathways such as PI3K and TOR [64], linking Myc with TOR-induced protein translation and highlighting a direct role of Myc in the regulation of translation in cancer [65].

### 2.5. Myc Control of Metabolism

In most tumors, c-Myc, Myc-N and Myc-L can be either amplified at their transcript level, or indirectly increased by growth factor signaling components, such as PI3K, RAS and by the beta-catenin/APC pathway [66,67,68,69,70]. At the metabolic level, it is well known that Myc overexpression is causing cancer cells to become independent of nutrients and it induces a metabolic switch able to activate pathways that foster tumor cells survival even in the absence of growth factors or amino acids [71,72,73,74]. Indeed, Myc in cancer cells favors the switch from oxidative phosphorylation to anaerobic glycolysis to produce lactate from glucose, through a process called “Warburg effect” [73,75]. In addition, Myc activates glutaminolysis, that uses the catabolism of glutamine and produces glutamate, to fuel the tricarboxylic acid (TCA) cycle through anaplerotic reactions necessary to produce the intermediates for cellular biosynthesis and essential amino acids [76].

Notably, in *Drosophila* these cellular metabolic events are highly conserved; indeed, we showed that, when overexpressed in a metabolic tissue like the fat body, Myc increases the expression of enzymes of the glycolytic flux and of lipid metabolism, resulting in increased levels of fats and glucose storage and consumption. Moreover, Myc in fat cells promoted survival pathways, like autophagy, allowing the animals to survive in low nutrient conditions [77,78]. In addition, animals expressing Myc in fat cells are bigger in size, phenocopying flies overexpressing Myc ubiquitously [33], thus suggesting a role for Myc in the control of non-autonomous pathways that regulate animal growth. Finally, also in *Drosophila,* Myc induces the expression of enzymes that control the metabolism of glutamine, a crucial amino acid in the regulation of glutaminolysis, another key pathway in the survival of cancer cells [77,79,80].

The specific role of Myc outlined in this chapter highlights its ability to induce processes that favor growth and cellular metabolism. As a consequence, Myc expressing cells are better fitted and acquire a more competitive behavior than cells that express lower levels of Myc. Indeed, Myc is among the genes that induce cell competition, a physiological process linked to cellular survival, that is described in the next chapter.

## 3. Cell Competition

As previously mentioned, Myc has a conserved role in the transcriptional regulation of genes involved in protein synthesis and ribosome biogenesis, including ribosomal protein genes. When mutated, these genes are classified as *Minutes* [19,37,38], and they were firstly described in *Drosophila* over 80 years ago [81]. All *Minute* mutations, which include at least 50 different genetic loci, have similar phenotypes: all are lethal when homozygous, and are associated with prolonged development and short size of the bristles or hairs present in the adult flies [82]. Heterozygous *Minute* mutants (*M/+*) show reduced viability and fertility, growth defects in eyes and wings, and small body size. Thus, one copy of a *Minute* gene is not sufficient to produce adequate protein amounts for normal development. In addition, heterozygous *M*/+ mutations abolish the ability of Myc expressing cells to act as supercompetitors [33,83]. Notably, in vertebrates, the oncogenic property of Myc was abolished in mice with a *Minute* mutation [84], suggesting for the first time a conserved function for Myc and cell competition in mice tumorigenesis.

Thus, cell competition is a fitness-sensing mechanism that is highly conserved in different organisms, involved in embryonic development, tissue regeneration and tumor progression [85]. According to this process, cells that are less fit compared to their neighbors (“loser” cells) are actively eliminated by the fitter cells (“winner” cells), that are, amongst other phenotypes, characterized by a higher proliferation rate [86,87,88].

### 3.1. Early Discoveries in Cell Competition

Cell competition was first described by Morata in the 1970s by studying the phenotype of *Drosophila Minute* mutant clones in the wing imaginal discs. He observed that *M/+* cells were growing slowly, and that in mosaic imaginal disc tissue they were always outcompeted by neighboring wild type cells, which eliminated them through apoptosis mediated by short-range interactions [89,90]. Thus, the first known trigger of cell competition was a difference in protein synthesis efficiency, resulting in differing growth rates between neighbor cells.

**Figure 4:**
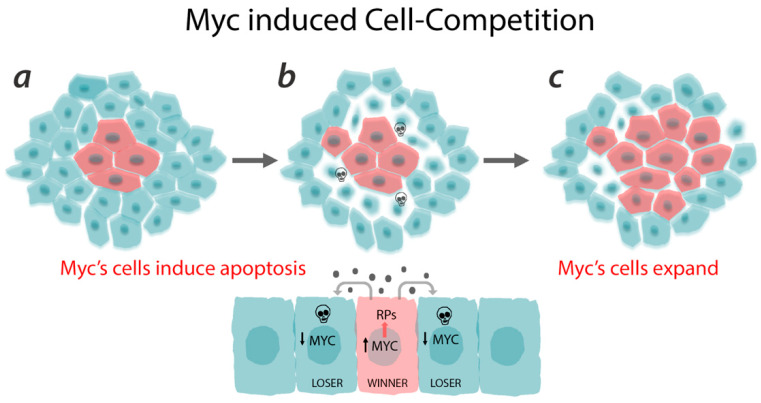
Schematic representation of Myc-induced cell competition. Myc cells are better fitted (***a***) and grow at the expenses of wild type cells that die of apoptosis (***b***) allowing Myc-expressing cells to grow and invade the field (***c***). In particular, here we focused on the ability of Myc-expressing cells (winner) to favor an optimal ribosomal function by inducing ribosomal proteins (RPs). Myc cells also release unknown short-range signal(s) that non-autonomously kill the neighboring cells, which also have a lower Myc level and a reduced ribosomal function (loser).

Several years later, Johnston et al. found that *dm*^*P*0^ mutant clones in *Drosophila* wing imaginal discs exhibited a similar behavior as the *Minute* tissues, with small cells that struggled to grow when compared to wild-type neighboring cells and died by apoptosis [32]. They identified this mechanism as cell competition, triggered by the growth advantage of wild type cells over Myc mutant cells. This effect also occurs when Myc is ectopically expressed in wild type cells (Figure 4) that then acquire the status of “supercompetitors” and outcompete wild type cells by inducing their death, leading to a complete repopulation of the field [33,83].

### 3.2. Cell Competition Mechanisms

Myc-induced cell competition is a process that is also conserved in vertebrates and is mediated by two important steps: first, it requires different levels of Myc between cells; second, cells have to be at close contact to trigger a signal that kills the neighboring cells (Figure 4). Indeed, elegant works using *Drosophila* cell cultures and human embryonic stem cells showed that competition could be fostered not only by short-range interactions, but also by non-autonomous mechanisms [91,92]. Senoo-Matsuda et al. have shown that conditioned medium generated from competitive co-cultures is able to induce cell death when incubated with loser cells, and cell proliferation when incubated with winner cells, suggesting that soluble molecules secreted by both cell types are involved in the competitive process [91].

In the past decade, other models have been suggested in order to define the mechanisms that drive or act downstream of cell competition, and along with Myc, a few other components have been characterized as drivers of the elimination of loser cells. One mechanism is the reduced capacity of the loser cells to uptake the secreted growth factor Decapentaplegic (Dpp), promoting the upregulation of the transcriptional repressor Brinker (Brk) that results in the induction of JNK-dependent apoptosis [93]. Some years later, Baker demonstrated that wild type cells are also able to eliminate neighbor loser cells by engulfment [94]. Moreno’s group showed the presence of a cell membrane protein called Flower (Fwe), present in winners or losers in different isoforms. Flower is induced by Myc in winner cells and is required for the activation of the protein Azot, that functions as cellular sensor for fitness and when reduced, induces JNK apoptotic cell death [95,96]. Another component is Sparc (secreted protein acidic and rich in cysteine), secreted on the cell membrane, that was found to inhibit caspase-3 activation in loser cells, protecting them from death [97]. More recently, Johnston et al. demonstrated that apoptosis in loser cells can be induced by the cytokine Spätzle (Spz), that binds to toll-related receptors (TRR) in loser cells to activate NFkB-dependent apoptosis [98,99].

Winner cells exhibit better fitness, and at the metabolic level Johnston et al. showed that Myc overexpressing cells, after coming into contact with wild type cells, undergo an increase in glycolysis with a mechanism that is dependent on p53 activity. In this context, p53 seems to work as a sensor for cell competition and is able to promote the supercompetitor status by controlling metabolism and proliferative capacity [79]. Therefore, cell competition is also regulated/influenced by the cellular metabolism. These metabolic changes and their link to cell fitness are an important and novel field of investigation that could reveal further similarities between cell competition induced by *Minute* or Myc and its relevance to cancer.

It is not yet clear whether and to what extent all the pathways above cooperate in driving the elimination of loser cells. It is also most likely that other factors are involved. Interestingly, these models are conserved between *Minute* and Myc dependent cell competition, suggesting that the selection of fitter cells, although driven by different factors, is regulated by common mechanisms.

### 3.3. Myc and Ribosomes as Drivers of Cell Fitness

*Minute* and Myc driven cell competition, through the described mechanisms, allows the selection of optimal cells and the elimination of inefficient ones, thus improving the overall fitness of a tissue. It is proposed that ribosomal functionality and the protein synthesis rate are crucial elements in determining cell fitness, and when these factors are perturbed competition events can be triggered between neighboring cells. The influence of Myc and ribosomes on cell fitness has been further investigated in recent years, analyzing in more detail their involvement in cell competition. The ability of Myc supercompetitor cells to prevail over losers depends also on the correct functionality of the ribosomal machinery [100], among which several Myc targets are present [21,37]. In particular, cells possessing four copies of *myc* have been shown to lose their ability to outcompete surrounding cells, and are even eliminated, after the induction of a heterozygous mutation on the *M(2)60E* gene [83], a Myc target that encodes for the ribosomal protein RpL19 [101].

Recent work from Baker’s lab showed that another ribosomal protein, RpS12, has a direct role in response to *Minute* driven cell competition. In loser cells, RpS12 was shown to activate the transcription factor Xrp1, a bZip-domain protein, that is responsible for a reduction in growth and in the overall translation rate, with a mechanism that is still unknown [100,102,103]. An unconventional role also emerged for RpS12, which is capable of globally regulating the protein synthesis rate, thus greatly influencing cell fitness. These results further highlight the role of ribosomal proteins in driving cell competition, and the potential collaborative role of Myc.

### 3.4. Physiological Role of Cell Competition

While few mechanisms involved in cell competition have been elucidated, the biological role of this process still needs to be clarified. Already in early observations made in *Drosophila*, these mechanisms emerged as essential for the optimal control of organismal development [104], and they are considered important to prevent organ overgrowth, thanks to the elimination of loser cells [33].

It has also been suggested that cell competition, both during development and in adult life, is a process by which genetic heterogeneity in tissues can be eliminated, ensuring that only optimal cells can survive and contribute to tissue functionality. Importantly, this hypothesis also comes from studies conducted in mouse models, where Claveria et al. reported a function for endogenous cell competition events in mammalian embryos. The authors demonstrated that Myc expression in epiblast cells is heterogeneous in early embryos, where cells with higher Myc levels can expand at the expense of cells with lower Myc levels that are eliminated by apoptosis, thereby insuring that only the most capable cells are selected to contribute to the new organism [105].

Endogenous competition events in vertebrates have also been described later in development and during organ formation. In mouse cardiomyocytes, cells exhibiting a reduced expression of the Myc isoform Myc-N [106] and of ribosomal genes are eliminated to ensure the optimal development of the heart [107].

An intense and novel field of study involves the hypothesis that cell competition is driven by a mechanical control of cell density to maintain tissue homeostasis [108]. This may involve the control of cell polarity by modification of the tumor suppressor genes *scribbled* (*scrib*), *discs large* (*dlg*), or *lethal giant larvae* (*lgl/Hgl-1*), responsible for controlling proper cellular polarization in tumor cells (discussed in the next paragraph). In support of this novel pathway, the component of the hemidesmosome, collagen XVII (COL17A1), that controls cellular force in the skin, has been associated with cell competition during ageing, suggesting that a control of fitness in the skin is part of a complex and physiological function for this organ during ageing [109]. Similarly, in the mouse epidermis, Myc-driven cell competition was shown to play a physiological role during skin development [110].

These data demonstrate that the mechanism of cell competition is evolutionarily conserved from the cells of *Drosophila* wing imaginal discs to mouse epidermis during development.

### 3.5. Cell Competition and Cancer

The role of cell competition in cancer prevention and development is still a matter of great discussion. It is known that some cell competition mechanisms have a tumor suppressive function, as they contribute to the elimination of defective cells with carcinogenic potential. In fact, cells with mutations in the above-mentioned tumor suppressor genes *scrib*, *dlg* or *lgl* are eliminated by adjacent wild type cells [111,112,113,114]. Several signaling pathways are involved in this anti-tumoral cell competition mechanism, such as Eiger/TNF-JNK, that are required for the induction of apoptosis in loser cells [115].

Of particular relevance is the role of Myc in the competitive behavior of *lgl* mutant cells, which present lower levels of Myc and die via JNK-dependent apoptosis. Moreover, the overexpression of Myc in those cells or in a *Minute* background allows the *lgl* mutant cells to bypass cell competition and acquire an overgrowth phenotype and invasive properties, suggesting that Myc levels and protein synthesis control the behavior of these cells [116]. Evidences for a similar regulatory behavior were obtained in the context of the Hippo (Hpo) tumor suppressor pathway, a conserved pathway between flies and humans that is important for the control of organ size and growth [117]. Myc is a target of Yki (YAP/TAZ fly’s orthologue), and it has been shown that different levels of Myc within the context of *Hpo* mutant clones are critical for determining whether cell competition between mutant cells and the neighboring wild type cells promotes or suppresses tumor growth [118,119].

Therefore, cell competition triggered by differences in cell fitness is a process that resembles tumor development in many aspects, and is often driven by oncogenes like Myc. It is possible that these mechanisms could also be involved in the initial stages of tumor development, and that pre-cancerous cells engage wild type cells in a competition to kill them through apoptosis, and then start invading the affected tissue [120,121,122]. Some evidence of a direct involvement of cell competition in tumor formation and development has been collected in *Drosophila,* and instances of Myc-driven cell competition have also been observed in human cancer cell lines [123,124].

## 4. Myc and Ribosomes in Cancer

### 4.1. The Contribution of Ribosomes in Cancer

Alterations in ribosome biogenesis due to mutations in genes encoding for ribosomal proteins is associated with the development of rare pathological diseases known as ribosomopathies, each of them characterized by specific defects in ribosome biogenesis and distinct clinical phenotypes. Although most often they involve bone marrow failure and/or craniofacial and skeletal defects, it is noteworthy that several ribosomopathies, among these Diamond–Blackfan anemia, Dyskeratosis congenita, Noonan syndrome, 5q-myelodysplastic syndrome, Treacher Collins syndrome and Shwachman–Diamond syndrome, have been associated with an increased risk of developing tumors [18,125,126,127,128,129,130]. Furthermore, nucleoli in malignant cells generally show an irregular shape, and increased number and size of nucleoli are frequently observed in malignant tumors including breast, prostate, uterine cervix, esophageal, gastrointestinal, colorectal and hepatocellular cancers. [5,131,132].

In general, failure of proper regulation/control of ribosomal biogenesis, both in normal growth conditions and in cellular stress conditions, may create a favorable environment for cancer initiation and progression. The upregulation of ribosome biogenesis has been shown to be essential for the tumorigenic process, and it is sustained by different signaling pathways, such as PI3K-AKT-mTOR, Myc and Ras-ERK, or by the inactivation of tumor suppressors like retinoblastoma (Rb) and p53 [5,65,133,134].

Counterintuitively, a decrease in the number of ribosomes may also contribute to tumorigenesis. Aberrant modifications of rRNA and reduction of ribosomes can decrease the rate of total protein synthesis and lead to a lower affinity of the translation machinery for specific mRNAs. This can in turn cause reduced production of tumor suppressor proteins, among other things [134]. An interesting example is X-linked dyskeratosis congenita (DKC), that is caused by mutations in the *DKC1* gene, encoding dyskerin. Previous studies have shown that these mutations can affect the affinity of ribosomes for translation of a specific subset of mRNAs, including p53 and p27 (both tumor suppressors) and two anti-apoptotic factors, XIAP/DIAP1 and Bcl-XL [135,136]. Thus, alterations in the expression levels of different components of the ribosomal formation could lead to heterogeneity of ribosomes, which can mediate translational reprogramming during cancer progression.

### 4.2. The Cooperation between Myc and Ribosomes in Cancer

A direct correlation between ribosome biogenesis and the ability of Myc to promote tumorigenesis was shown first by Barna and co-workers, who demonstrated that loss of one single allele of *RpL24* or *RpL38* in Eµ-Myc mice decreased the incidence of lymphoma by 20% and delayed tumor onset, along with the re-establishment of accurate translational control and genome stability. Thus, in this case a modest reduction in the expression of a single ribosomal protein gene was sufficient to reduce Myc ability to promote tumor onset [84]. Another stronger indication that increased ribosome biogenesis can be a direct cause of malignant transformation was provided by Devlin et al., who have demonstrated that synergistically targeting ribosome biogenesis and mRNA translation increased survival in Myc-driven lymphoma [137]. The relevance of ribosome biogenesis in Myc driven tumor formation was also outlined by Hald et al., where the synthetic reduction of ribosomal synthesis, in patients affected by MYCN amplified neuroblastomas, repressed the growth of the tumors [138].

Another puzzle is the mechanism(s) that underlies the elevated incidence of tumors, such as acute myeloid leukemia, colon carcinoma, and osteogenic sarcoma, in patients with inherited ribosomopathies such as Diamond–Blackfan anemia (DBA) caused by heterozygous mutations in RP genes including *RPL5*, *RPL11*, and to a lesser extent *RPL19* and *RPS26*, or for *RPS14* haploinsufficiency found in myelodysplastic syndromes (MDS) [139]. In DBA it was shown that nucleolar stress due to mutations in RPL5 and RPL11 results in their binding and inhibition of MDM2, which subsequently causes in p53 accumulation [140]. Recently, Myc was shown to stabilize the RPL5–RPL11–5S rRNA complex suggesting it has a role in the control of p53 activation [141]. However, we can speculate that the RPL5-L11 and MDM2-p53 axis may be induced by Myc as the result of a feedback loop required to prevent Myc-induced tumorigenesis.

Ribosomal proteins may also be playing an important role in metastasis, as it was recently shown by that overexpression of RPL15 in mice increases their growth, opening a completely new perspective on the role of the ribosome in the field of metastasis [142].

Thus, investigating ribosomal assembling and protein translation, and how abnormal ribosomes bypass quality control surveillance, are important steps to better understand the onset of cancer, and in particular of Myc-induced tumorigenesis.

## 5. Conclusions

In conclusion, many mutations in ribosomal proteins have been shown to cause a predisposition to cancer, including some linked to Myc activation. However, a clear mechanism of their function has not been elucidated. We can certainly hypothesize that with the discovery of Myc-induced cell competition in *Drosophila*, and because of the many similarities in the mechanisms that control ribosomal biogenesis, the use of *Drosophila*’s genetic tools may help in better understanding what mechanisms drive tumor progression induced by ribosome biogenesis and Myc.

*Drosophila* has emerged as a valuable model to study cancer [143]. Indeed, sophisticated genetic tools give the opportunity to manipulate the expression of cancer-related genes in epithelial tissues (i.e., the imaginal discs). Moreover, most of the genes and pathways analyzed in humans are conserved in *Drosophila*, thus it is possible to confidently recapitulate various aspects characteristic of human cancers, including proliferative capacity, invasiveness and metabolic adaptation typical of tumor cells to support cell growth. Therefore, it is expected that this model will continue to significantly contribute to the investigation of the link between Myc, ribosome biogenesis, and tumor development.

Moreover, since many Myc functions are preserved in *Drosophila*, this model has generally proved useful for investigating the role of this protein in tumorigenesis. In fact, several oncogenic pathways result in stabilization or overexpression of Myc, causing robust cell growth that can lead to cancer development in flies as well as in vertebrates [143]. Fascinatingly, these evolutionarily conserved functions of Myc to control mass and metabolism are among the reasons for the selective advantage of growth of epithelial cells described in cell competition. In addition, the aberrant effects of Myc in regulating global tumor translation and its function in controlling nucleolar size and number, hallmarks of cancer, are conserved also in the tiny fruit fly.

Many elements are still missing to understand how truly relevant the role of Myc and of cell competition is in the tumorigenic process, and possibly the precise mechanisms by which this occurs. Although a few mutations in ribosomal proteins have been associated with specific tumors and with the activation of Myc, how these effects are coordinated is still not clear. With this review we outlined the evidence of Myc in controlling ribosome biogenesis with its role in cell competition, a hypothesis that may connect the ability of Myc to initiate tumorigenesis by promoting cell competition during the initial step of field cancerization.

## Abbreviations

snoRNP	small nucleolar ribonucleoproteins
DREF	DNA replication-related element binding factor
TFIIB	Transcription factor II B
TRAPP	Trafficking protein particle complex
GCN5	Histone acetyltransferase GCN5
TAFs	TATA box-binding protein-associated factor-s
TIF-IA	Transcription intermediary factor-1A
UBF/SL-1	Nucleolar transcription factor 1/
TIF-IB	Transcription intermediary factor-1B
Xrp1	Transcription factor/bZip DNA binding protein
